# Supplementary material for: How effective were Australian Quarantine Stations in mitigating transmission aboard ships during the influenza pandemic of 1918-19?
Source: PLoS Comput Biol. 2023 Nov 27;19(11):e1011656. doi: 10.1371/journal.pcbi.1011656 (PMC10703403; doi:10.1371/journal.pcbi.1011656)
Supplement: Attachment — Submitted filename: Alahakoon et al. - review.pdf [file pcbi.1011656.s003.pdf]

Alahakoon et al. analyze outbreaks of 1918 pandemic influenza on four Australian ships using a Bayesian hierarchical model. The hierarchical modeling framework offers promise in estimating parameters of dynamic epidemiological models (e.g., many predictive COVID-19 models fit states/locations independently), and this manuscript has the potential to demonstrate the technique in an epidemiological setting. Furthermore, biological insights from the analysis may be useful. However, as it currently stands, this manuscript does not clearly motivate or address either of these potential contributions (methodological or epidemiological). My comments are as follows:

1. The manuscript would benefit from a primary research question or guiding purpose, that is relevant for the scientific community today. For example, some potential questions that I believe are currently being addressed include:
    a. Variability in transmission rates between different ships and different types of passengers (crew/passenger)
    b. Effect of quarantine on reducing ship outbreak size

   Yet, these questions are not clearly motivated in the introduction as being important. For example, what do estimated transmission rates tell us about the current biology/control of influenza or other infectious diseases? Why might one study the effect of quarantine on outbreak size within a ship? Aren't we instead interested in the effects of quarantine on controlling spread in the population more generally?

2. The model structure, fitting procedure, and subsequent analyses are complex and impressive. However, the important details of these complexities aren't clear in the methods. I recognize that much of the information is already provided in the supplemental material, but it may benefit readers to include a version in the main text as well.
    a. Currently details about ship-specific data are scattered throughout section 2.3 and the supplement. I think it would be helpful to describe these features of the data in more detail at the outset (in section 2.2), especially as it seems available data guided choices about model structure. This context would be useful before reading sections 2.3 and 2.4. (e.g., line 192 says "addressing the challenges of the available data", but these challenges are not clearly outlined).
    b. There are modeling assumptions that are stated in the main text, but the reader is not given justification.
        i. Model structure: Why are there multiple A classes? What is the purpose of the C classes, rather than these individuals moving to R? Is it to model deaths? If so, is there evidence to suggest that such individuals are no longer infectious?
        ii. Line 206: "all other model parameters are ship specific" – Why does it make sense for some biological parameters (e.g., recovery rate) to be ship-specific, rather than constant across ships? Relatedly, why is transmission rate the only parameter with hierarchical structure?
    c. More detail about the "specified distance criteria and tolerance values" (lines 210-211) would be useful context for the reader.

d. The sampled, resampled, and conditional resampled paths are central to understanding the results and figures. Yet currently, there is only one sentence in the methods. Elaboration and clarification on this portion of the analysis is needed.

3. All estimated intervention effects (brown histograms) contain a significant density below 0, suggesting quarantine increased new infections. This is contrary to expectation and should be explained. The authors may consider the possibility presented by Kaminsky et al. 2019.

Additional comments:

4. Line 153-154: The stochastic simulation framework (Gillespie, I believe) should be mentioned in the main text.
5. Line 194-195: "all other parameters across the groups were equal" – is "the groups" referring to asymptomatic vs. symptomatic, or crew vs. passenger? If this is referring to crew vs. passengers, it would be useful to remove the i subscript from those parameters in Figure 2.
6. Line 223: "(Step 2 of the two-step algorithm)" I do not see the first or second step defined in the methods.
7. Figure 4: this figure is very complex, which makes it difficult to see what the key takeaway is.
8. Line 299-305: What is the purpose of evaluating "a ship that had similar properties as *Medic*"?
9. Line 367-368: "This framework was instrumental in improving the overall parameter estimates in comparison to studying each outbreak independently" – Although potentially addressed in Figure S11, I do not see how this was shown in the current methods/results in the main text. It would be interesting and useful to discuss.
10. Line 374-376: discussion of future applications of hierarchical model estimation framework – It seems that the hierarchical framework could be useful in dynamical epidemiological models more generally. It might be useful for the authors to provide additional discussion on the benefits/limitations of such an approach (e.g., computational time, accuracy of estimates, etc.)
11. All figure legends need to be more specific.

References

Kaminsky, Joshua, Lindsay T. Keegan, C. Jessica E. Metcalf, and Justin Lessler. 2019. "Perfect Counterfactuals for Epidemic Simulations." *Philosophical Transactions of the Royal Society B: Biological Sciences* 374 (1776): 20180279. https://doi.org/10.1098/rstb.2018.0279.
