# Supplementary material for: How effective were Australian Quarantine Stations in mitigating transmission aboard ships during the influenza pandemic of 1918-19?
Source: PLoS Comput Biol. 2023 Nov 27;19(11):e1011656. doi: 10.1371/journal.pcbi.1011656 (PMC10703403; doi:10.1371/journal.pcbi.1011656)
Supplement: Attachment — Submitted filename: PCOMPBIOL-D-23-00364-Review.docx [file pcbi.1011656.s004.docx]

The authors model the transmission of influenza on 4 ships, The Medic, Boonah, Devon and Manuka, from an epidemic recorded in 1918 by hospitals aboard each individual ships, as well as while docked quarantine stations. An extended SEIR-type multi-compartmental model was fit to the different ships, using Bayesian hierarchical modeling to estimate the parameters that best describe the transmission dynamics between each compartment. The effects of non-pharmaceutical interventions (quarantining and removal of individuals showing elevated temperatures or symptoms) were also assessed in terms of infections and deaths averted. While the approach is generally robust and rationale for modeling assumptions are provided, outstanding limitations remain before this paper can be considered for publication.

**Main Text Comments**:

1. A clearer description of the data is needed. It is not clear how so many compartments that distinguishes between asymptomatic or pre-symptomatic individuals, and mild and/or severe illnesses is possible. Do the data contain these types of granular assessments by the hospital personnel both on the ships and the quarantine stations? I know this is difficult to capture even in the present-day. Please provided a description of the data that clearly illustrates the capture of these metrics (a/pre-symptomatic, mild vs. severe infection, daily temperature assessments, etc…). Otherwise, the model seems likely over-specified/complex and likely has parameter identifiability limitations (see bullet 3 below).
2. Why were these 4 particular ships chosen to model out of the other 79 that had reported outbreaks? The “qualitative” description you provide on lines 121 – 125 doesn’t provide details as to how many ships even had these data available, or what metrics you used to arrive at the 4 ships chosen for modeling (completeness? Availability? Other quality metrics?). Please provide a clearer explanation, evaluations methods used, summary statistics and other quantitative measures used to arrive at your final population of 4 ships. Arguably, including more ships would provide more precision in the hierarchical modeling framework, but this point is not discussed by the authors.
3. Figure 2, p. 10: Structural and practical considerations for parameter identifiability not provided given the data. Non-unique solutions may exist if identifiability is an issue, and this limitation is not thoroughly explained. For example, see reference: "Global identifiability of nonlinear models of biological systems" Audoly et al., 2001. This seems particularly troubling given the bi-modal nature of the hyperparameter distributions (Figure S10), and the generally wide variance of projected paths (Figure 3), posterior distributions of transmission rates (Figure 4), etc.
4. I need clarification: for the counterfactual scenario sample path generation, were the appropriate eta_i and zeta_i parameters (corresponding to disembarkation) removed from the model and refit to calculate infections/deaths averted, correct? As described, I’m not sure if I follow the counterfactual path generation. Please comment/clarify any changes to the model for the sampled path vs. conditional path generation.
5. Please provide a line that represents the “median” paths generated from the fitted curves to the data. This will make interpretation of the model fit easier for readers (and also allow you to calculate some goodness-of-fit metrics).
6. I think the author puts the poor fit of the Boonah model to the observed data too lightly. In reality, the model is likely wrong/not representative of the influenza infection process occurring. Were other parsimonious compartmental models or modeling assumptions tested to obtain a better fit? The authors do not describe other efforts to better fit the data, even with this over-specified model. The hypothesis posed by the authors on lines 315 – 325 are speculative, at best.
7. A major limitation of including the Manuka in the hierarchical modeling scheme is that the data is only available after day 8, meaning that many of the disease transmission rates are borrowed from the other ships (clearly seen in the Supp2, Figure S11, for both the Medic and Manuka ships). This limitation should be clearly included in the discussion, and also used as an imperative to further provide quantitative metrics of why these 4 ships were included in the study, while the other 79 were not. Data availability/summary of completeness would be a start.
8. No quantitative goodness-of-fit evaluations, plots or metrics are provided (only graphs with observed vs. paths, which would benefit from adding a median line as suggested earlier). AIC/BIC for alternative models tested could also be informative, if indeed the compartmental models that were settled on were explored beforehand (simpler/complex models with more/fewer compartments).

**Supplement 1 Comments**:

1. Consider putting breaks in the bar graphs in order not to compress small counts towards the y-axis (Figure S1, S2).

**Supplement 2 Comments:**

1. Prior correlation matrix subscripts on upper triangle seem off…? Please check.
2. Please comment on the impact of the hierarchical model on the Passenger -> Crew transmission rates on the Medic ship (Figure S11). It seems to have a disproportionate impact compared to the other ships/transmission rates. This is also seen for the Manuka, but it is understood that there is less data available (only day 8 onward) for the time-series data (as written on lines 259-260).
3. S3.2 – summary statistics – it’s written that influenza cases could be “confirmed or uncomfirmed”. Please describe in the main text how this was done (temperature, symptom criteria checklist…? As written, it’s not clear).
4. Is it possible to overlay the posterior parameter distributions for the common parameters included in all models (for each ship)? In other words, collapsing/overlaying panels in Figure S4.2 for the same parameter if it is included in multiple models. Per Figure 2, I understand some models/compartments are unique for a ship, but many of them are in all models. This would allow to easily qualitatively compare the variance/precision of each parameter for each ship, collectively.
