# Supplementary material for: How effective were Australian Quarantine Stations in mitigating transmission aboard ships during the influenza pandemic of 1918-19?
Source: PLoS Comput Biol. 2023 Nov 27;19(11):e1011656. doi: 10.1371/journal.pcbi.1011656 (PMC10703403; doi:10.1371/journal.pcbi.1011656)
Supplement: Attachment — Submitted filename: Reviewer_response.docx [file pcbi.1011656.s005.docx]

James McCaw

School of Mathematics and Statistics,

University of Melbourne,

Melbourne,

Australia.

Email: [jamesm@unimelb.edu.au](mailto:jamesm@unimelb.edu.au)

September 4, 2023

**Manuscript Number:** **PCOMPBIOL-D-23-00364)**

Dear Associate Professor Virginia Pitzer,

Thank you for your consideration of our manuscript *How effective were Australian Quarantine Stations in mitigating transmission aboard ships during the influenza pandemic of 1918-19?* and the opportunity to submit a revised version addressing the reviewers’ comments.

In responding to the reviewers’ comments below, we have also taken the opportunity to fix some minor grammatical errors and made some further minor changes for clarity. These are all documented in the track-changes version of the manuscript.

We would be happy to answer any further questions that you may have and look forward to hearing your decision.

Kind regards,

James McCaw

(On behalf of all the authors)

**Reviewer #1;**

The authors model the transmission of influenza on 4 ships, The Medic, Boonah, Devon and Manuka, from an epidemic recorded in 1918 by hospitals aboard each individual ships, as well as while docked quarantine stations. An extended SEIR-type multi-compartmental model was fit to the different ships, using Bayesian hierarchical modeling to estimate the parameters that best describe the transmission dynamics between each compartment. The effects of non-pharmaceutical interventions (quarantining and removal of individuals showing elevated temperatures or symptoms) were also assessed in terms of infections and deaths averted. While the approach is generally robust and rationale for modeling assumptions are provided, outstanding limitations remain before this paper can be considered for publication.

**Main Text Comments**:

1. A clearer description of the data is needed. It is not clear how so many compartments that distinguishes between asymptomatic or pre-symptomatic individuals, and mild and/or severe illnesses is possible. Do the data contain these types of granular assessments by the hospital personnel both on the ships and the quarantine stations? I know this is difficult to capture even in the present-day. Please provided a description of the data that clearly illustrates the capture of these metrics (a/pre-symptomatic, mild vs. severe infection, daily temperature assessments, etc…). Otherwise, the model seems likely over-specified/complex and likely has parameter identifiability limitations (see bullet 3 below).

In our original submission we have provided an explanation of the assumptions we made with respect to each ship. The process that was undertaken prior to the development of the model, and its application to the data, are detailed in the records provided through the GitHub repository as referenced in our manuscript. Nonetheless, we have updated the manuscript to draw the reader’s attention to this data pre-processing step. The Methods describe our decision-making process, and we now explicitly direct the reader to the GitHub repository (in green below) for detailed annotations for each recorded case.

Supplementary Material 1 S1.1 includes details of the GitHub repository where annotations and assumptions for each ship record can be found.

Generally, when the records of these ships mentioned removal of infectious/infected individuals as ‘mild cases’, ‘serious cases’ and ‘cot/convalescent cases’, we referred to them as ‘mild infections’, ‘severe infections’, and ‘recovering individuals’ respectively. In instances where these exact words were lacking from the original records, we have made assumptions as described in the Methods section. For example, if an individual was admitted to the hospital on board, they have been counted as having had a severe infection.

In the record of *Medic,* the medical officer or the medical personnel from the Quarantine Station documented if the cases were mild, severe, or recovering. We have now included an additional figure (see below) in the supplementary material (and referenced in the main text) to distinguish between removal of mild and severe infections. The infections that took place on board the ship were all either recorded as severe cases (in the record) or assumed to be so as part of our data pre-processing steps.

In the record of *Boonah,* there is no differentiation between mild and severe cases and therefore, we did not include separate compartments for mild and severe cases (see original submission Figure 2). Removal of convalescent cases is described, and so included in the model.

For *Devon* and *Manuka*, the records do not describe removal of convalescent individuals and so no flows for disembarkation are included in the model (see original submission Figure 2).

Furthermore, the records of *Medic* and *Boonah* describe the removal of “healthy’’ individuals from the ship to the shore. As we have described in the manuscript (Section 2.3), we account for the disembarkation of these people, taken to be those *not* in compartments with symptoms (so including susceptible, exposed, asymptomatic, pre-symptomatic and recovered individuals).

Given the detailed data available (as described in the manuscript, and summarised above), we believe that our mathematical model is of an appropriate complexity. The additional compartments (convalescent and mild infectious, etc) included for some ships but not others were based on the level of detail available in the data for that ship. Flows to model disembarkation of infectious, infected and healthy individuals were also included according to the detail available in the data.

Note that where we modelled disembarkations (where data are available in a ship’s record), they were incorporated into our ABC parameter estimation framework as additional summary statistics (Supplementary Material 2 S3.2)

While these methods were described in the original submission (paragraph 6 of Section 2.3, which directs the reader to further details in the supplementary material), in light of the reviewer’s queries we now provide additional detail in the main text:

Our model structures for each group (passengers, crew) are shown in Figure 2. When modeling the dynamics of *Medic*, we used the full model structure (Figure 2: *Medic*) based on the detailed description of the outbreak in the original source material and our annotation of it (refer to Supplementary Material 1 S1). This ensured that our model’s complexity (in terms of the compartmental structure) was commensurate with the level of detail contained in the ship record, including for the removal of infectious, infected and healthy individuals. For *Boonah* (Figure 2: *Boonah*) and *Devon* and *Manuka* (Figure 2: *Devon* and *Manuka*), for which the original source material provides less information, we did not use the full model structure. Rather, we only included the flow through mildly infectious compartments as the records do not differentiate between severe and mild infections. Similarly, we did not model the removal of healthy, infectious, or infected individuals for *Devon* and *Manuka* as there were no records of removals for these ships. Supplementary 1 S4 describes the detailed dynamics for each of the ships and the corresponding modelling assumptions we made in relation to the available data*.*

Finally, regarding the reviewer’s comment on likely challenges with parameter identifiability, we of course understand the general point being raised, and address it in full below (point 3). Here, we note that 1) the underlying data, as described above, supports the use of models with the given complexity; and 2) one of our primary goals in this work is to study counterfactual scenarios (in which removal of individuals from the ships does not occur) which does not rely on unique identification of parameters.

a) Why were these 4 particular ships chosen to model out of the other 79 that had reported outbreaks? The “qualitative” description you provide on lines 121 – 125 doesn’t provide details as to how many ships even had these data available, or what metrics you used to arrive at the 4 ships chosen for modeling (completeness? Availability? Other quality metrics?). Please provide a clearer explanation, evaluations methods used, summary statistics and other quantitative measures used to arrive at your final population of 4 ships.

Most ships’ records describe that they had infections on board. However, only a few had sufficient details of the dynamics of the outbreaks to warrant a dynamic-model based analysis. We have added the following details under the section Data:

For each of the seventy-nine ships that travelled overseas and on which passengers and/or crew acquired influenza, we first examined each ship’s record to identify those that reported a major outbreak during their voyage. As a threshold, we chose only those ships that reported at least 20 cases during the voyage (22 ships). Of those 22 ships, most records had key information such as the total epidemic size (grouped by crew, civilian, and/or troop), total population size, source of infection, and port of arrival in Australia. However, only 11 of the 22 ship records included daily prevalence or incidence data, which is necessary for conducting a time-series analysis. The records for five of these 11 ships, while describing the time-series in the textual description of the outbreak(s), did not record the total number of infections by group. A further two ships had outbreaks that were recorded as only having taken place among one of the three groups (crew, civilian, troops), so were excluded from analyses given concern that data on infections in other groups may be systematically missing. This left four of the 79 ships available for analysis (Boonah, Medic, Devon and Manuka).

These four shipboard outbreaks had detailed time-series data *on daily new case numbers, daily prevalence, incidence, the final size of the epidemic and sufficient qualitative information to formulate a suitable transmission model for the outbreak.*

b) Arguably, including more ships would provide more precision in the hierarchical modeling framework, but this point is not discussed by the authors.

We certainly agree in principle that more time-series data from other ships would support our analyses and improve our estimates for both individual ships and for the hyper-parameters describing the (ship)population level parameters. We already discuss the potential benefit of including additional time-series data from other ships to improve parameter estimates under a hierarchal framework in the Discussion. We have now explained that most of the outbreak data are inadequate for this purpose (previous comment). We have made additional edits to the paragraph that describes these points:

We note that improved estimates and insights into the outbreaks on board ships during the 1918 influenza pandemic may be obtained if more outbreaks with suitable time-series data were studied. In the absence of that data, the only other potentially suitable outbreaks that may be of interest are those that took place onboard *Niagara, Nestor, Ceramic*, and *Makura* which provide only final size data or only group-aggregated time-series data. Further investigation of how inclusion of that additional data may help (or hinder) analysis is left as a topic for future study.

3. Figure 2, p. 10: Structural and practical considerations for parameter identifiability not provided given the data. Non-unique solutions may exist if identifiability is an issue, and this limitation is not thoroughly explained. For example, see reference: "Global identifiability of nonlinear models of biological systems" Audoly et al., 2001. This seems particularly troubling given the bi-modal nature of the hyperparameter distributions (Figure S10), and the generally wide variance of projected paths (Figure 3), posterior distributions of transmission rates (Figure 4), etc.

This is a concern that is implicit in fields such as forecasting (epidemic forecasting, weather forecasting etc., where Bayesian (particle) filtering is used to identify regions of parameter space consistent with past data, and where evaluation is based on predictive performance (Yang, W., Karspeck, A., & Shaman, J. (2014)). It has also been described in settings similar to those here, including by the authors (see papers such as Ivo Siekmann, James Sneyd, Edmund J Crampin (2012), Campbell, P. T., McCaw, J. M., & McVernon, J. (2015) and Yan, A. W., Zaloumis, S. G., Simpson, J. A., & McCaw, J. M. (2019).)

We have been careful to not make any claim of uniqueness throughout the manuscript unless warranted by careful evaluation of the posterior distributions. However, as described in detail in our response to the first point, our approach to developing the models (for each of the ships) was to ensure that the biological fidelity/complexity of the model was commensurate with the detail provided in the primary data. This of course does not mean we are guaranteed to have unique solutions for all parameters, and it is clear that we do not. But our approach to align model complexity with data complexity does support the choices we have made. With only four ships, of course our hyperparameter distributions are broad, and we have been very careful to not overinterpret these results. Related to this, the reviewer makes a comment about a bi-modal distribution (Figure S10) but we don’t see from the figure that there is any evidence for this statement. Figure S11 shows that we do obtain tighter estimates for certain parameters (e.g., transmission parameters on the *Medic*) under our hierarchical analysis (in comparison to the non-hierarchical analyses) supporting our approach.

Equally importantly, our focus on simulated paths and counterfactuals, and not just parameter estimates, mitigates concerns on this front. Reconstruction of these epidemics (which is not contingent on identification of unique solutions) is a primary outcome of this work, as it is what is required to support the counterfactual analyses.

4. I need clarification: for the counterfactual scenario sample path generation, were the appropriate eta_i and zeta_i parameters (corresponding to disembarkation) removed from the model and refit to calculate infections/deaths averted, correct? As described, I’m not sure if I follow the counterfactual path generation. Please comment/clarify any changes to the model for the sampled path vs. conditional path generation.

When the counterfactuals of no disembarkations are considered, the parameters relating to disembarkations must be zero. But the model is not refit as we are exploring what may have happened on board that ship in the absence of disembarkations, not how one might attempt to describe the outbreak that occurred if disembarkations were ignored (a study for which we do not believe there would be any scientific justification). The model (with disembarkation rates set to zero) was re-simulated. We have updated our description in Section 2.6.

When the counterfactual with no disembarkation is considered, we simulated from the models for the *Medic* and *Boonah* with the rates of removal of healthy, infectious or infected set to zero.

We compared each outbreak with the counterfactual of having no individuals disembark after arriving at port. These quarantine measures were studied for two research questions:

1) By how much were the interventions on the ships *Medic* and *Boonah* estimated to have changed the final size of the epidemic.
2) Were the disembarkations expected to have reduced the total number of infections on ships/voyages with similar properties to *Medic* and *Boonah*.

We studied (1) by generating conditional re-sampled paths and (2) by generating re-sampled paths.

1) Conditional re-sampled paths:

These are paths that were accepted under the Approximate Bayesian Computation (ABC) acceptance/ rejection criteria. When carrying out the parameter estimation using the ABC two-step algorithm we have introduced previously (Alahakoon et al., 2022), during the second step of this algorithm, we generate sample paths for both the original model (that is, with disembarkations) and the counterfactual path. Whenever the sample path from the original model is accepted, we also accepted the corresponding counterfactual path.

2) Re-sampled paths:

These paths were similarly generated by using the estimated parameters for the original model and the counterfactual model, without applying the ABC acceptance/rejection criteria. This is similar to a posterior predictive check.

As all these details are included in the manuscript, and further details are included in the Supplementary material, we have not made any changes to the manuscript.

5. Please provide a line that represents the “median” paths generated from the fitted curves to the data. This will make interpretation of the model fit easier for readers (and also allow you to calculate some goodness-of-fit metrics).

Thank you for this suggestion which we have considered carefully. The wider literature on time-series analyses, including in epidemiological modelling, contains many misleading visual representations of posterior densities. Pointwise median curves – often presented as they are straightforward to calculate, do not provide a suitable visual guide for time-series data and in some circumstances can be actively misleading (Lofgren, E. T. (2012)). However, curvewise analyses, for both the median and intervals, are suitable. We have adjusted Figure 3 to include the curve-wise intervals (50\%, 75\%, and 95\%) of conditional re-sampled paths (in green) and re-sampled paths (in purple) under the hierarchical estimation algorithm. Black lines are the curvewise median paths, and the blue dot-dashed lines represent the data.

As well as this change, we have also included a sentence in the manuscript (Section 3) stating that the reader may refer to a figure presenting the trajectories of conditional re-sampled and re-sampled paths for these ships for further reference.

See Supplementary Material 2 S4 for full trajectories of the conditional re-sampled and re-sampled paths for these ships.

Furthermore, we have included the removal of infectious/infected for Medic and Boonah based on the re-sampled paths to ascertain the predictive power of the models in terms of removals. These plots can be found in Supplement 2. We have now included the following sentence in the main manuscript (Section 3):

See Supplementary Material 2 S5 for time-series plots of infectious/infected and healthy individuals aboard Medic and Boonah based on re-sampled paths.

6. I think the author puts the poor fit of the Boonah model to the observed data too lightly. In reality, the model is likely wrong/not representative of the influenza infection process occurring. Were other parsimonious compartmental models or modeling assumptions tested to obtain a better fit? The authors do not describe other efforts to better fit the data, even with this over-specified model. The hypothesis posed by the authors on lines 315 – 325 are speculative, at best.

As we have explained in the Discussion, we suspect that there may have been a change (an increase) in case ascertainment once *Boonah* arrived at the quarantine station. This discrepancy appears only in the tail-end of the epidemic. This change in the dynamics is not supported by any epidemiological or public health documentation or other descriptive information in the primary record (please refer to the primary historical record and our annotations on it in our GitHub repository). Of course, we cannot exclude that there was some other event or interaction that is not structurally captured by our model. We refer the reader back to our response to their point 1 and the process we undertook to develop the compartmental structure, and to *a priori* determine if its degree of complexity was suitable. Clearly, our chosen model (both its compartmental structure and our modelled observation process) for the *Boonah* has not captured the abovementioned phenomenon. While additional compartments may provide sufficient flexibility to capture it, we argue that such a model would be over-specified and motivated only for the purpose of capturing/fitting this feature in the data. As our model formulation, assumptions and calibration depend on the available data and information only, we are only able to hypothesize/speculate on other possibilities.

7. A major limitation of including the Manuka in the hierarchical modeling scheme is that the data is only available after day 8, meaning that many of the disease transmission rates are borrowed from the other ships (clearly seen in the Supp2, Figure S11, for both the Medic and Manuka ships). This limitation should be clearly included in the discussion, and also used as an imperative to further provide quantitative metrics of why these 4 ships were included in the study, while the other 79 were not. Data availability/summary of completeness would be a start.

We disagree. Rather than a major limitation of the study, it is a clear example of the advantage of using a hierarchical framework, where partial (but informative) data can be incorporated into an analysis. This general point has been well explored in the literature. We have implemented our analysis through a hierarchical analysis in order for information sharing to be possible among the four ships. Furthermore, even though we do not have any *time series* data for *Manuka* until day 8, we do have details on *how many* infections took place by the time the ship reached day 8. We have now specifically included this advantage in the discussion:

Furthermore, while the record of *Manuka* had no time-series data until day 8, it does include the total number of infections that had occurred by day 8. The information sharing properties of the hierarchical modelling framework provided significant benefit here, leading to improved parameter estimates for *Manuka* (as seen by comparing independent and hierarchical estimation frameworks in Figure S12 in Supplementary Material 2)*.*

We have explained in the previous comments, that although there were 79 outbreaks, only four had sufficiently detailed time-series data to be included in the analyses.

8. No quantitative goodness-of-fit evaluations, plots or metrics are provided (only graphs with observed vs. paths, which would benefit from adding a median line as suggested earlier). AIC/BIC for alternative models tested could also be informative, if indeed the compartmental models that were settled on were explored beforehand (simpler/complex models with more/fewer compartments).

We emphasize that we are using Bayesian methods. We have now provided the curve-wise interval and have already addressed model complexity issues raised by this reviewer in the previous comments. Posterior-predictive checks are considered best practice (Berkhof, J., Van Mechelen, I., & Hoijtink, H. (2000)). Posterior predictive checks: Principles and discussion. *Computational Statistics*, *15*, 337-354) and qualitative goodness of fit evaluations are not typically reported. AIC/BIC are unsuitable as they do not provide evaluation of the quality of the full posterior. They are neither Bayesian nor measures for predictive evaluation.

While noting that our approach was to develop models that we *a priori* anticipated to have suitable complexity, we did evaluate the performance of a (far) simpler model. A stochastic SIR model (details are available in our GitHub repository for those interested) did not predict the data well enough to be considered as an alternative model.

**Supplement 1 Comments**:

1. Consider putting breaks in the bar graphs in order not to compress small counts towards the y-axis (Figure S1, S2).

Axis breaks were already applied to these figures. However, we have now increased the number of breaks. Furthermore, we have done the same for Figure 1 and S3 for consistency.

**Supplement 2 Comments:**

1. Prior correlation matrix subscripts on upper triangle seem off…? Please check.

Thank you, we have checked them, and they are correct.

2. Please comment on the impact of the hierarchical model on the Passenger -> Crew transmission rates on the Medic ship (Figure S11). It seems to have a disproportionate impact compared to the other ships/transmission rates. This is also seen for the Manuka, but it is understood that there is less data available (only day 8 onward) for the time-series data (as written on lines 259-260).

*Medic*, unlike *Devon* and *Boonah*, does not contain the daily incidence/ prevalence data of specific groups (please refer to the data explanations we have included in the GitHub folder or the snapshot of Medic in the man text). We only have the total number of infectious individuals of crew and total (crew and passenger) time-series daily prevalence data. As seen in the figure the reviewer is referring to, when the outbreak on board the Medic was considered by itself, the estimation was poor. However, under the hierarchical model, estimates were greatly improved (owing to the sharing of information between outbreaks). Furthermore, we point out that not all the ships contain similar types of data. They contain different types of data (some totals, some time-series by group, some time-series by both groups, etc.), and *Manuka* specifically stands out as it contains significant amounts of missing data. Because of the data sharing properties of the hierarchical model, we observe that parameters that would otherwise have been poorly estimated are now well estimated. In short, these are all benefits of using a hierarchical model. However, the focus of this study is not to specify the benefits (which are well documented in the literature) of using hierarchical models, but to make use of them, and therefore, we have not made any changes to the manuscript.

3. S3.2 – summary statistics – it’s written that influenza cases could be “confirmed or uncomfirmed”. Please describe in the main text how this was done (temperature, symptom criteria checklist…? As written, it’s not clear).

Unfortunately, how a case was determined to be either “confirmed” or “unconfirmed” is not clearly explained in the original record, so we cannot address this (and we suggest there is no need to, as the reader can review our annotated copy of the original record if interested). That said, we note that it is difficult to distinguish between an influenza-like disease (say, the common cold) and a mild case of influenza without proper testing, and of course in 1918 there were no tests (the causative agent, the influenza virus, was not to be discovered for another few decades). We note the following explanation on the diagnosis in the record:

In our study, we assume that if cases were confirmed, they may either be influenza cases or influenza-like diseases. For this very reason, we did not include records of ships that only mentioned “unconfirmed cases” (see our explanation on the data). In the manuscript, we have included *“Furthermore, some of the ships’ recorded cases of disease were not confirmed as influenza although they had influenza-like symptoms (high fever, pneumonia, etc.).”.* In addition to this sentence, we have now included the following sentence:

These unconfirmed cases may be considered as individuals who showed fever-like symptoms.

4. Is it possible to overlay the posterior parameter distributions for the common parameters included in all models (for each ship)? In other words, collapsing/overlaying panels in Figure S4.2 for the same parameter if it is included in multiple models. Per Figure 2, I understand some models/compartments are unique for a ship, but many of them are in all models. This would allow to easily qualitatively compare the variance/precision of each parameter for each ship, collectively.

We have now overlayed the posterior distributions of the common parameters of all the four ships as below:

**Reviewer #2**

Thank you for the opportunity to review this manuscript. This manuscript looks at four outbreaks of Spanish flu on ships that were returning to Australia from WWI in 1918. The analysis estimates transmission rates amongst those on board and looks at whether maritime quarantine, implemented at that time, was effective (e.g. removing infectious, convalescent, healthy after arrival at quarantine station).

This manuscript is well written, the paper has a sensible and logical flow, the methods are reproducible, and conclusions follow from results. This work would be of interest to the epidemiological modelling readership and perhaps beyond given this is an interesting case study of historical data and deploys hierarchical ABC for parameter estimation. This was a pleasure to read and I have very few comments – I recommend this manuscript for publication as it has been submitted.

Some minor comments are below – I would suggest these minor changes to help the reader but I leave their inclusion to the authors’ discretion.

**Minor comments**

1. Line 194: “rates of transmission between asymptomatic (βijA) and symptomatic infectious (βijI) are the same” -> perhaps rephrase to use “from” instead of ”between” since the transmission is between these and the susceptible class.

Thank you for pointing this out, the necessary changes have been made.

2. Line 210: “sample paths” and “simulated paths” are used (L234) – consider aligning language throughout.

Thank you, throughout the manuscript, we now refer to all these paths as simulated paths.

3. Figure 7 : “capitalize” station

Thank you, typos in this figure as well as in Figure 6 (similar mistake) are now fixed.

4. Introduction: We demonstrated that generally, in the ships that transported troops, the transmission rates within crew and passengers were higher than those between the crew and passengers. -> suggest specifying “passengers (civilians and troops)”, otherwise it is lost on a reader how troops factor into the statement.

Thank you, we made the necessary changes.

5. Acknowledgements: please clarify “across 32 clusters”

Here, the computer clusters refer to virtual computers on the Nectar cloud. We have included that in the acknowledgement as below:

Unless otherwise mentioned, computations were carried out in MATLAB or R across 32 clusters (32 virtual computers).

6. References: Several references need proper nouns capitalized (e.g. “Western Samoa”, “Spain”, etc).

Thank you for pointing this out, we have made the necessary changes.

7. S2.1: 10. R_i = Recovered state of asymptomatic individuals. (I believe this is symptomatic individuals)

Yes, thank you, the changes have been made.

8. S1: Can the perturbation kernel for the ship-specific ABCs be specified explicitly?

They are normal distributions with the standard deviation estimated from the particles of the previous generation. We have made the following changes:

We used normal densities with standard deviations estimated from the particles from the previous generations as the perturbation kernel when the ABC-SMC algorithm was run for each outbreak independently.

9. S1: How were the tolerance values for the summary statistics determined?

These were identified by running a basic ABC algorithm with very high tolerance values and displaying the relationship with parameters and the distance metrics. This is similar, but an extension to our work Alahakoon et al (2022). We now include this in the introduction paragraph in Supplementary Material 2:

Suitable tolerance values were chosen by running basic ABC algorithms with high tolerance values and displaying the sample of parameter values and distance metrics (similar to the approach described in Alahakoon et al (2022)).

10. Code: I would suggest consolidating descriptions of the contents of each folder in the repo to all be in the main README file instead of distributed throughout the different folders.

All the descriptions are now included in the main README file.

11. Code: I would suggest having a short description of how another researcher should run the code (i.e. very briefly state first, second, third steps; I believe all analyses are intended to be called from their respective folders).

We have now included the folder structure, and which files to run in the main README file.

12. Code: It seems that all data necessary for reproducing the analysis is provided in the repository – it is worth stating this explicitly.

Yes, we have now included all the necessary are data available to reproduce the results and in the main README file as well as in acknowledgement.

A snapshot of the GitHub Readme file:

**Reviewer #3**

Alahakoon et al. analyze outbreaks of 1918 pandemic influenza on four Australian ships using a Bayesian hierarchical model. The hierarchical modeling framework offers promise in estimating parameters of dynamic epidemiological models (e.g., many predictive COVID-19 models fit states/locations independently), and this manuscript has the potential to demonstrate the technique in an epidemiological setting. Furthermore, biological insights from the analysis may be useful. However, as it currently stands, this manuscript does not clearly motivate or address either of these potential contributions (methodological or epidemiological). My comments are as follows:

1. The manuscript would benefit from a primary research question or guiding purpose, that is relevant for the scientific community today. For example, some potential questions that I believe are currently being addressed include: a. Variability in transmission rates between different ships and different types of passengers (crew/passenger)
b. Effect of quarantine on reducing ship outbreak size. Yet, these questions are not clearly motivated in the introduction as being important. For example, what do estimated transmission rates tell us about the current biology/control of influenza or other infectious diseases? Why might one study the effect of quarantine on outbreak size within a ship? Aren’t we instead interested in the effects of quarantine on controlling spread in the population more generally?

We believe, along with other reviewers, that there is inherent value in studying past outbreaks of infectious diseases. Our study has prompted us to develop innovative models (in terms of compartmental structure) and test our recently developed Bayesian statistical inference algorithms. The vast literature on the study of the 1918-19 epidemic provides clear evidence for the value of studies such as ours.

From an epidemiological and public health perspective, we were primarily interested in estimating the key epidemiological parameters associated with the outbreaks that took place aboard these ships and identifying if Australian Quarantine Stations were effective in mitigating transmission on board the ships (as was intended based on the disembarkation of healthy individuals, as described in the primary source records). It would be interesting to investigate if quarantine stations were effective in mitigating importation and onwards transmission into the (Australian) population, However, this cannot be addressed with the data we have (which concerns only disease onboard the ships) Furthermore, if quarantine stations were in fact effective in mitigating transmission on board the ships we would expect that would indirectly reduce the probability of seeding infections into the (Australian) community in general.

For these reasons, we argue that our research questions are both interesting and important.

We included the following sentences (in green) into the Introduction.

We were primarily interested in estimating the key epidemiological parameters associated with these outbreaks that can provide improved insights to influenza outbreaks on board ships. Furthermore, we investigated whether Australian Quarantine Stations were beneficial in mitigating transmission on board these ships.

Consequently, *we modeled each outbreak using a ship-specific stochastic two-group (crew and passenger) metapopulation model that captured the dynamics of a ship's journey and its time at an Australian Quarantine Station until the outbreak died out. We used a Bayesian hierarchical modeling approach to estimate model parameters. We demonstrated that generally, in the ships that transported troops, the transmission rates within crew and passengers were higher than those between the crew and passengers.* We further calculated that the basic reproduction numbers, R_0_, for *Medic*, *Boonah*, *Devon*, and *Manuka* were 6.38 (median) [(3.96, 11.06) quartile], 2.53 [2.07, 3.25], 4.46 [2.78, 8.97], and 2.45 [1.87, 3.96] respectively. These numbers were generally high and consistent with most settings where the populations were closed and isolated. *Furthermore, we demonstrated through counterfactual analysis, that, the removal of individuals from aboard the Medic reduced the spread of infection. If all persons had remained aboard, we estimated an additional 18 (median) [(5, 31) quartile range] infections would have occurred. In contrast, removals from the Boonah were estimated to have had a negligible impact on the outbreak aboard the vessel*. Overall, we concluded that Quarantine Stations in Australia provided varied benefits, depending on the epidemiological status of the ship at the time of quarantine.

2. The model structure, fitting procedure, and subsequent analyses are complex and impressive. However, the important details of these complexities aren’t clear in the methods. I recognize that much of the information is already provided in the supplemental material, but it may benefit readers to include a version in the main text as well.

a. Currently details about ship-specific data are scattered throughout section 2.3 and the supplement. I think it would be helpful to describe these features of the data in more detail at the outset (in section 2.2), especially as it seems available data guided choices about model structure. This context would be useful before reading sections 2.3 and 2.4. (e.g., line 192 says “addressing the challenges of the available data”, but these challenges are not clearly outlined).

We have displayed the available data (Figure 1, and in the supplement) and have explained what additional data are available. We have included more details about the available data as per Reviewer 1’s comments. We believe it will not be an effective use of the main text of the manuscript to include all the details of each ship (which are long and complex and are most appropriately described in the supplement as well as the GitHub folder). All the key details for the available data are included in Section 2.2 (Data) and explanatory details that were essential for model formulation are given in Section 2.3 (modeling disease outbreaks).

However, we agree that we omitted to include key details regarding the removal of infectious, infected, and healthy people on board in Section 2.2. These details are now included (in green below):

*Of the Medic's 989 population, there were 829 troops. The rest of the population consisted of 4 civilians and 156 crew. Boonah's population consisted of 916 troops and 164 crew, a total of 1080. Of Devon’s 1096 population, 920 were troops, 66 were civilians, and 110 were passengers. Manuka's record does not mention the transportation of troops. Of its population of 203, 95 were crew and 108 were assumed to be civilians.* The records of *Medic*, *Boonah*, and *Manuka* document the prevalence of cases up to the time they arrived at the quarantine stations. Out of them, only *Medic* and *Boonah* describe the removal of infectious, infected, and healthy people from the ships to be quarantined and/or treated. *Table 1 provides a summary of the details of each ship’s population, voyage, quarantine details, and outbreak characteristics. Figure 1 shows a snapshot of the data available for the Medic}. Supplementary Material 1 S1.2 presents illustrations for the other three ships.*

b. There are modeling assumptions that are stated in the main text, but the reader is not given justification.

The justifications are given in the supplementary material. We have already included in the main text (Section 2.4) that all the justifications are included in the supplementary material. Again, we believe it would disrupt the flow of the main text of the manuscript to provide additional details, and that they are better explained in detail in the supplement.

i. Model structure: Why are there multiple A classes? What is the purpose of the C classes, rather than these individuals moving to R? Is it to model deaths? If so, is there evidence to suggest that such individuals are no longer infectious?

This has been raised by reviewer 1 and we have addressed it in our response to them. The reason for using the *C* class is not just to model the deaths, but more importantly to model the removal of infected (recovering, but no longer infectious) individuals. The reason for using two *A* classes is to make sure that the mean times spent on each branch of the model are comparable.

ii. Line 206: “all other model parameters are ship specific” – Why does it make sense for some biological parameters (e.g., recovery rate) to be ship-specific, rather than constant across ships? Relatedly, why is transmission rate the only parameter with hierarchical structure?

It is a modeling choice (i.e., an assumption) we made for transmission rates to be under a hierarchical model (see details on the estimation framework) and we have demonstrated that this choice was sufficient (but neither necessary nor a unique requirement) to enabled improved inferences compared to fitting each ship independently. As is evident from the available data, each of the four ships has different characteristics and it would have been a strong assumption to model all the ships to have identical epidemiological parameters (including for example for the recovery rate). Nonetheless, we agree that an alternative statistical structure, in which additional or different parameters were modelled under a hierarchy may also be a valid approach. No changes have been made to the manuscript.

c. More detail about the “specified distance criteria and tolerance values” (lines 210-211) would be useful context for the reader.

We have used multiple summary statistics and tolerance values depending on the available data of the four ships. We have dedicated most of the Supplementary material 2 for this purpose as the procedure is complex and must be explained in detail. However, in order to provide more context, we have adjusted the main text:

*specified distance criteria and tolerance values* depending on the available data of the ships.

d. The sampled, resampled, and conditional resampled paths are central to understanding the results and figures. Yet currently, there is only one sentence in the methods. Elaboration and clarification on this portion of the analysis is needed.
As we have explained in our response to Reviewer 1, these paths are merely accepted paths via ABC parameter estimation. Predicted paths are what are produced using posterior predictive checking, which we have re-named in our context to improve clarity for the readers. We believe sufficient details are already provided and no changes have been made to the manuscript.

3. All estimated intervention effects (brown histograms) contain a significant density below 0, suggesting quarantine increased new infections. This is contrary to expectation and should be explained. The authors may consider the possibility presented by Kaminsky et al. 2019.

We do not consider this observation to be ‘contrary to expectation’ or in any way surprising as our models are stochastic. We agree that the paper by Kaminsky et al. 2019 explains that this is a possibility due to stochastic simulations. When observed and counterfactual path are generated per estimated parameter set of a model, we ensured that the path generated up to the time of a ship arriving at a quarantine station in both observed and counterfactual settings was identical (see the supplementary material for details of how this was achieved), and then generated the observed and counterfactual paths from that time onwards. Therefore, our results are based on appropriately computed observations and counterfactuals after arriving at the quarantine station. If the counterfactual path died out earlier than the observed path with interventions or happened to result in fewer infections, which is of course a possibility due to the stochastic nature of our model, this may result in a value of less than zero for the difference (as illustrated in the histogram). We have now included these explanations after *Medic:*

Note that the histograms (panels (C) and (F) of Figure 6 and the ones illustrated consequently), show some values for the difference in the total number of infections between paths under the main and counterfactual analyses to be less than zero, indicating that the counterfactual paths may have died out earlier or otherwise resulted in fewer infections. This is entirely expected due to the stochastic nature of the models.

**Additional comments**

4. Line 153-154: The stochastic simulation framework (Gillespie, I believe) should be mentioned in the main text.

We have now made the following changes.

*Supplementary Material 1 S3 provides more details on how we simulated paths* (using the Doob-Gillespie (Gillespie, 1977; Doob, 1945) algorithm) *with this model structure.*

5. Line 194-195: “all other parameters across the groups were equal” – is “the groups” referring to asymptomatic vs. symptomatic, or crew vs. passenger? If this is referring to crew vs. passengers, it would be useful to remove the i subscript from those parameters in Figure 2.

The groups are passengers and crew.

The compartmental diagram (Figure 2) is shown for only one group, and therefore labelling both the compartments and associated rates, for which we make no assumptions on similarity or difference across groups, with an index (subscript *i*) is important. Because transmission occurs both within and between groups, two indices (*i*, *j*) are required for the transmission parameters.

Note that the line “all other parameters across the groups were equal”, a modeling assumption, comes *after* we have introduced the models in Figure 2.

We have adjusted the caption to Figure 2:

“The compartments of the models are shown for group *i* (*i=1 (crew); i=2(passengers)*). The population is separated into those who are susceptible (S_i_), exposed (E_i_), asymptomatic (A_1i,_ A_2i_), pre-symptomatically infectious (I_iP_), mild (M_i_) and severely (I_iS_) infectious, mild and severely infected (C_Mi_, C_i_), severely infectious/infected and quarantined (I_iQ_,C_iQ_), and recovered (R_Ai_,R_i_,A_iQ_). The force of infection, $\lambda_i$ has terms for both within- and between- group transmission.”

6. Line 223: “(Step 2 of the two-step algorithm)” I do not see the first or second step defined in the methods.

The two-step algorithm is the algorithm we used for parameter estimation, which has two steps. We have cited this work and is beyond the scope of this work. However, we have now specifically mentioned the purpose of Step 2.

(Step 2 of the two-step algorithm which enables estimation of the ship-specific parameters under the hierarchical framework)

7. Figure 4: this figure is very complex, which makes it difficult to see what the key takeaway is.

Thank you, we have simplified Figure 4 as below:

8. Line 299-305: What is the purpose of evaluating “a ship that had similar properties as *Medic*”?

This would allow us to analyse and make inferences about the ships that were similar to as *Medic* but do not have sufficient information and data to model them. However, we believe the appropriate explanation should not be restricted to *Medic*, but also for *Boonah* as we have done the analysis for both ships. Therefore, we included an explanation (in green) below in Section 2.5 where we have described the analysis with counterfactuals.

*Next, we asked if the disembarkations would have been expected to reduce the total infections on ships/voyages with properties as estimated for Medic and Boonah.* The reason we were interested in this question is that in the records of Cumpston (1919), there were ships of similar characteristics as *Medic* and *Boonah*, but they could not be included in the analysis due to lack of sufficient data to model them. Therefore, *to answer this question, we simulated paths with interventions and counterfactual paths from the joint posteriors. Unlike when addressing the first question, the ABC acceptance/rejection step was not applied. This method is similar to the posterior predictive check (Berkhof, Van Mechelen, and Hoijtink,2000), and in this study, we will describe these simulated paths as re-sampled paths.*

9. Line 367-368: “This framework was instrumental in improving the overall parameter estimates in comparison to studying each outbreak independently” – Although potentially addressed in Figure S11, I do not see how this was shown in the current methods/results in the main text. It would be interesting and useful to discuss.

We have now included figures (Supplementary Material 2 and shown below) comparing the predictive power of independent and hierarchical estimation frameworks based on re-sampled paths. These figures illustrate that even by only including transmission rates in the hierarchical framework, we are able to make improved predictions (by peak, range of the intervals, etc.), with comparison to an independent estimation. Accordingly, we have now referenced these figures in the manuscript as below (in green):

*This framework was instrumental in improving the overall parameter estimates in comparison to studying each outbreak independently (refer to Supplementary Material 2 S6 for a comparison of curve-wise intervals based on re-sampled paths and see Alahakoon et al (2022) for an explanation).*

10. Line 374-376: discussion of future applications of hierarchical model estimation framework – It seems that the hierarchical framework could be useful in dynamical epidemiological models more generally. It might be useful for the authors to provide additional discussion on the benefits/limitations of such an approach (e.g., computational time, accuracy of estimates, etc.)

The use of hierarchical models is a well-established theory in statistics (see the references below) and their advantages are well documented. Use of dynamical models for malaria within a hierarchical framework had been studied by our co-author James McCaw. Our previous study, Alahakoon et al (2022), which we have already cited in the manuscript has studied the usage of stochastic epidemic models within a hierarchical framework. Computational time, accuracy and efficiency issues are usually dependent on the complexity of the dynamic model and the available data. We have included the following paragraph at the end of the Discussion.

Hierarchical models are, however, generally useful for improving parameter estimates, addressing missing and incomplete data issues, and allowing inferences on data at multiple levels (population and sub-population level) (Gelman and Hill, 2006; Kruschke, 2014; Gelman, Carlin, Stern, Dunson, Vehtari, and Rubin, 2013). Furthermore, it is possible to use a hierarchical framework when the outbreaks are modelled as dynamical models (Alahakoon et al., 2022a; Alahakoon, McCaw, and Taylor, 2022b). They have been previously applied to deterministic dynamical modeling settings in malaria (Cao, Collins, Zaloumis, Wattanakul, Tarning,Simpson, McCarthy, and McCaw, 2019). However, computational time, efficiency and accuracy depend on the complexity of the dynamical model and the available data.

11. All figure legends need to be more specific.

We believe the reviewer is referring to the captions of the figures. We have made necessary changes to the Figures.

References

Kaminsky, Joshua, Lindsay T. Keegan, C. Jessica E. Metcalf, and Justin Lessler. 2019. “Perfect Counterfactuals for Epidemic Simulations.” *Philosophical Transactions of the Royal Society B: Biological Sciences* 374 (1776): 20180279. https://doi.org/10.1098/rstb.2018.0279.
