# Supplementary material for: How effective were Australian Quarantine Stations in mitigating transmission aboard ships during the influenza pandemic of 1918-19?
Source: PLoS Comput Biol. 2023 Nov 27;19(11):e1011656. doi: 10.1371/journal.pcbi.1011656 (PMC10703403; doi:10.1371/journal.pcbi.1011656)
Supplement: Attachment — Submitted filename: PCOMPBIOL-D-23-00364-Review_Resubmission.docx [file pcbi.1011656.s006.docx]

These comments correspond to the initial comments provided by the reviewer (and subsequently, the author’s responses to them).

1. The addition of text providing further explanation/context for the details of the data is sufficient. The figure (and related figures) could be more clear by removing the orange dots at the top of the bars, and instead increase the font to clearly communicate the quantity represented by the bars.
2. This makes the decision making of which data to include much more clear. As a final improvement, including this logic in an “attrition diagram/table” would make it abundantly clear to the readers, but this decision rests with the editor/authors.
3. I stand corrected, the bimodal distributions are indeed Figure S11 from Supplement 2 (not S10, as I initially wrote). To me, this essentially makes any counterfactual estimation (and inference) a moot point and more of an exploratory exercise. In their response, the author attempts to disregard the importance the lack of precision of these parameters on the “…Reconstruction of these epidemics…as is the counterfactual analyses.”
4. Thank you for these clarifications.
5. The authors indeed have provided what I intended to communicate, thank you.
6. I think the author somewhat agrees with my worry of poor fit/wrong model structure, but also tries to offer some justifications/explanations. In fact, they seem to have answered this question later on (see comment #8) as they tried to perform modeling with a more parsimonious SIR model (fewer compartments), but the predictions were no good. It could be more simple to say on line 435… that “It is clear from the deviations of the predicted paths that our model could not accurately model all influences of disease transmission rates aboard the Boonah. For instance….”, or something more to that tone (stressing limitations), and then go into the various explanations you offer.
7. I think the authors misinterpreted my concern here. The symptomatic rates of infection *cannot* be modeled without assumptions regarding the relationships/similarities (or *data interchangeability)* between Medic/Boonah/Devon (as is clearly shown in Figure 3). The strengths of hierarchical models are also their limitation: how similar were there other ships (and their passengers/crew/troops transmission rates) to the 3 that actually had information? Whether these parameter estimates are “improved” or just simply incorrect is a limitation I would like listed as a limitation of the study. Thank you.
8. Listing a few reasons why your current model structure was chosen (over the simpler ones you considered that are included on GitHub) would go a long way for readers of the text. For instance, posterior predictive checks of simpler models in the supplement would help, as the author has suggested.

Supplement 1 Comments

1. Breaks (not tick marks, as the author may have interpreted) in the y-axis are *not* provided in either the current or prior versions of the graph, and the bars are still significantly “squished”. The quality of the figure could be improved but does not impact the interpretation of the data.

Supplement 2 comments

1. Prior correlation matrix subscripts: please check this again (top of page 2, Supplement 2). I see the top row subscripts seem off (should all read "1[x]", where "1" indicates the row "[x]" is the column. Currently, the first row reads [1, p12, p22, p21], which is inconsistent with the rest of the correlation matrix.
2. Addressed above
3. Ok
4. This figure is great, thank you for including it.
