# Supplementary material for: How effective were Australian Quarantine Stations in mitigating transmission aboard ships during the influenza pandemic of 1918-19?
Source: PLoS Comput Biol. 2023 Nov 27;19(11):e1011656. doi: 10.1371/journal.pcbi.1011656 (PMC10703403; doi:10.1371/journal.pcbi.1011656)
Supplement: Attachment — Submitted filename: response-submitted.docx [file pcbi.1011656.s007.docx]

James McCaw

School of Mathematics and Statistics,

University of Melbourne,

Melbourne,

Australia.

Email: [jamesm@unimelb.edu.au](mailto:jamesm@unimelb.edu.au)

October 24, 2023

**Manuscript Number:** **PCOMPBIOL-D-23-00364)**

Dear Associate Professor Virginia Pitzer and Professor Claudio José Struchiner,

Thank you for your consideration of our manuscript *How effective were Australian Quarantine Stations in mitigating transmission aboard ships during the influenza pandemic of 1918-19?* and the opportunity to submit a revised version addressing the reviewer’s comments.

All changes made to the manuscript are documented in the track-changes version and detailed in this revision letter.

We would be happy to answer any further questions that you may have and look forward to hearing your decision.

Kind regards,

James McCaw

(On behalf of all the authors)

These comments correspond to the initial comments provided by the reviewer (and subsequently, the author’s responses to them).

1. The addition of text providing further explanation/context for the details of the data is sufficient. The figure (and related figures) could be more clear by removing the orange dots at the top of the bars, and instead increase the font to clearly communicate the quantity represented by the bars.

Thank you, we have now revised the figure and included an additional table (below) to clearly specify the qualitative (figure) and quantitative (table) nature of the removals after arriving at the Quarantine Station. This replaces the original figure in the main text (now Figure 2 (B)). We have removed this from the supplement to avoid repetition of figures. For consistency, a similar figure (and a corresponding table) are now included for *Boonah* (as *Boonah* also included details of removals of individuals) in the Supplementary Material S1.

Number of individuals who were removed from the Medic after arriving at the Quarantine Station and their disease status.

| Day | Healthy removals | Total infectious/infected  Removals | Mild cases | Severe cases | Unknown (mild/severe)  cases |
| --- | --- | --- | --- | --- | --- |
| 20 | 0 | 82 | 50 | 32 | 0 |
| 21 | 0 | 57 | 43 | 14 | 0 |
| 22 | 34 | 0 | 0 | 0 | 0 |
| 23 | 151 | 0 | 0 | 0 | 0 |
| 24 | 0 | 38 | 0 | 38 | 0 |
| 25 | 0 | 108 | 0 | 0 | 108 |

2. This makes the decision making of which data to include much more clear. As a final improvement, including this logic in an “attrition diagram/table” would make it abundantly clear to the readers, but this decision rests with the editor/authors.

We have now included an attrition diagram below to the manuscript in Section 2.2 Data. We have also referenced this figure in the manuscript as below:

See Figure 1 for a summary of this outbreak selection procedure.

Attrition diagram that presents the process of choosing four outbreaks to study.

3. I stand corrected, the bimodal distributions are indeed Figure S11 from Supplement 2 (not S10, as I initially wrote). To me, this essentially makes any counterfactual estimation (and inference) a moot point and more of an exploratory exercise. In their response, the author attempts to disregard the importance the lack of precision of these parameters on the “…Reconstruction of these epidemics…as is the counterfactual analyses.”

While some marginal posterior distributions in Figure S11 are certainly wide, they are from the independent (i.e., non-hierarchical) analysis and so are not relevant to the counterfactual analyses. The posteriors from the hierarchical analysis are generally tighter and, in our view show very little, if any sign, of bi-modality. In any case, we know of no statistical issue for the counterfactual analyses (based on sampling from the joint-posterior of course, not separately from each of the marginals). Our approach embraces the uncertainty in our knowledge, and we demonstrate where we can (and cannot) identify statistically robust differences in outcomes based on the interventions that were applied.

4. Thank you for these clarifications.
5. The authors indeed have provided what I intended to communicate, thank you.
6. I think the author somewhat agrees with my worry of poor fit/wrong model structure, but also tries to offer some justifications/explanations. In fact, they seem to have answered this question later on (see comment #8) as they tried to perform modeling with a more parsimonious SIR model (fewer compartments), but the predictions were no good. It could be more simple to say on line 435… that “It is clear from the deviations of the predicted paths that our model could not accurately model all influences of disease transmission rates aboard the Boonah. For instance….”, or something more to that tone (stressing limitations), and then go into the various explanations you offer.

We have adjusted the discussion:

However, the conditional re-sampled paths of *Boonah* display a clear deviation from the data from following the 17^th^ day, the day of arrival at the Quarantine Station. While this indicates that our model has almost certainly not captured all of the influences of disease transmission dynamics aboard the *Boonah* and/or the observation process for the outbreak, there was no further information within the historical record to justify introduction of a more complex model. We further note that once a ship arrived at the Quarantine Station, the ship’s population was tested, and therefore, a greater fraction of infectious cases than what had been identified during the voyage may have been identified, increasing case ascertainment. These additional cases may also have included infectious cases with mild symptoms. This provides one plausible explanation for the observed deviation between the data and our model’s output at the tail-end of the course of the epidemic. However, this hypothesised explanation cannot be evaluated as our model (appropriately) does not capture nor differentiate between mild and severely infectious persons since this aspect of the epidemic was not described in the record.

7. I think the authors misinterpreted my concern here. The symptomatic rates of infection *cannot* be modeled without assumptions regarding the relationships/similarities (or *data interchangeability)* between Medic/Boonah/Devon (as is clearly shown in Figure 3). The strengths of hierarchical models are also their limitation: how similar were there other ships (and their passengers/crew/troops transmission rates) to the 3 that actually had information? Whether these parameter estimates are “improved” or just simply incorrect is a limitation I would like listed as a limitation of the study. Thank you.

Thank you for your comment, we agree that it is important to consider the similarity of the ships.

Within a hierarchical framework, please note that data interchangeability does not take place. Instead, data sharing takes place through *parameter exchangeability* (textbooks such as Gelman et al (2013) discuss this in detail*).* The form of this is the conditional prior distribution, which we have modelled using a normal distribution with hyper-parameters for the mean and standard deviation. If the variability between ship-specific parameters is high, then the standard deviation at the hyper-parametric level would be expected to be high. In such situations, it is possible to obtain posterior distributions for the ship-specific parameters that have wider credible intervals with incorrect estimates. We direct the reviewer to our recent work on this (Alahakoon, P., McCaw, J. M., & Taylor, P. G. (2022)).

We refer to this reviewer’s comment (#2 in the previous queries) and our response on choosing 4 outbreaks (including the *Manuka*) out of 79 outbreaks. We ensured that the outbreaks we chose had broadly similar data structures, despite the missing data of *Manuka*. More importantly, these outbreaks took place at very similar times (October-November 1918), and it is reasonable to assume that the same 1918 influenza variant was prevalent on board each of the ships. Each ship’s crew and passengers (including troops) can be assumed to share broadly similar demographic characteristics (although the records do not document this). Therefore, it is reasonable to assume that these outbreaks shared biological, epidemiological, and behavioral characteristics and consequently, may have similar transmission rates. We believe this justifies our adoption of a hierarchical framework. From this, we conclude (but of course cannot guarantee) that for the *Manuka,* whose record had missing data, our analyses benefit from information sharing with *Medic, Boonah,* and *Devon.*

We have now included these clarifications the manuscript as below in green:

*We used a Bayesian hierarchical modeling framework [27–30] to study the outbreaks that took place on board the ships. Given that the ships sailed at similar times (October-November 1918) and that it is reasonable to assume that 1) all four ships were impacted by the same virus (the 1918 influenza virus); and 2) the demographic characteristics of those aboard the ships were similar, our choice of a hierarchical analysis is appropriate. Under this framework, we modeled each outbreak using the stochastic models introduced in Section 2.3. …*

Finally, we note the inclusion through the previous round of review of figures and analyses in Supplementary Material 2 that compare the predictive power of the independent and hierarchical estimation frameworks. We have made a minor adjustment to the text describing these analyses that are pertinent to the query just addressed:

*This framework was instrumental in improving the overall parameter estimates in comparison to studying each outbreak independently (refer to Supplementary Material 2 S6 for a comparison of curve-wise intervals based on re-sampled paths and see Alahakoon et al (2022) for an explanation).*

8. Listing a few reasons why your current model structure was chosen (over the simpler ones you considered that are included on GitHub) would go a long way for readers of the text. For instance, posterior predictive checks of simpler models in the supplement would help, as the author has suggested.

This figure was already included in the GitHub, however, now we have also included this figure (below) in the Supplementary material.

Accordingly, we added the following section to Supplementary Material 2 for *Medic* as it was the ship for which we used the most complex model.

**Use of other model structures for *Medic***

To test whether other simple model structures were suitable for *Medic* we also undertook our analysis with a simple two-group SEAIR-type model in which we excluded the compartments for those classified as mild infectious (M__i_), mild recovering (C__Mi_), and severely infected and recovering (C__i_)). This model failed to accurately predict the removal of infectious, infected, and healthy individuals (Figure S22).

Boxplots of removal of infectious, infected and healthy individuals of Medic based on a simpler
model that does not take into account compartments M_i_, C_Mi,_ and C_i_ based on re-sampled paths. The observed values are represented as orange triangles.

We have also referenced this in the manuscript as below (in green) in Section 2.3-Modeling disease outbreaks:

*Our model structures for each group (passengers, crew) are shown in Figure 2. When modeling the dynamics of Medic, we used the full model structure (Figure 2: Medic) based on the detailed description of the outbreak in the original source material and our annotation of it (refer to Supplementary Material 1 S1* and *Figure S22 for details on the unsatisfactory performance of an alternative simpler model*).

**Supplement 1 Comments**

1. Breaks (not tick marks, as the author may have interpreted) in the y-axis are *not* provided in either the current or prior versions of the graph, and the bars are still significantly “squished”. The quality of the figure could be improved but does not impact the interpretation of the data.

Please note that the bars look “squished” due to the large variability between the population numbers (example 16 vs. 916) and adding breaks may not suffice. However, as a better solution, we have now plotted these numbers on a log10 scale (see below). For consistency we have made similar changes to the snapshots of other ships as well (in the manuscript for *Medic* and in the Supplement for *Manuka* and *Devon*).

Supplement 2 comments

1. Prior correlation matrix subscripts: please check this again (top of page 2, Supplement 2). I see the top row subscripts seem off (should all read "1[x]", where "1" indicates the row "[x]" is the column. Currently, the first row reads [1, p12, p22, p21], which is inconsistent with the rest of the correlation matrix.

Thank you, they were in fact typos. We have now corrected this as below for the first row:

[1 p12 p13 p14]

2. Addressed above
3. Ok
4. This figure is great, thank you for including it.
